# Mild Uncoupling of Mitochondria Synergistically Enhances Senolytic Specificity and Sensitivity of BH3 Mimetics

**DOI:** 10.59368/agingbio.20240022

**Published:** 2024-02-20

**Authors:** Edward P. Fielder, Abbas Ishaq, Evon Low, Joseph A. Laws, Aisha Calista, Jemma Castle, Thomas von Zglinicki, Satomi Miwa

**Affiliations:** 1 Newcastle University Biosciences Institute, Campus for Ageing and Vitality, Newcastle University, Newcastle upon Tyne, United Kingdom; 3 Newcastle University Centre for Cancer, Newcastle University, Newcastle upon Tyne, United Kingdom

## Abstract

Despite immense potential as anti-aging interventions, applications of current senolytics are limited due to low sensitivity and specificity. We demonstrate the specific loss of complex I-linked coupled respiration and the inability to maintain mitochondrial membrane potential upon respiratory stimulation as a specific vulnerability of senescent cells. Further decreasing the mitochondrial membrane potential of senescent cells with a mitochondrial uncoupler synergistically enhances the in vitro senolytic efficacy of BH3 mimetic drugs, including Navitoclax, by up to two orders of magnitude, whereas non-senescent cells remain unaffected. Moreover, a short-term intervention combining the mitochondrial uncoupler BAM15 with Navitoclax at a dose two orders of magnitude lower than typically used rescues radiation-induced premature aging in an in vivo mouse model, as demonstrated by reduced frailty and improved cognitive function for at least eight months. Our study shows compromised mitochondrial functional capacity is a senescence-specific vulnerability that can be targeted by mild uncoupling in vitro and in vivo.

## Introduction

Together with apoptosis, cellular senescence is one of the major cellular stress responses with pleiotropic consequences during development and aging. While cell senescence contributes to organ remodeling and wound healing, accumulation of senescent cells during aging has been proven as a major cause of many, if not all, major age-associated diseases and disabilities, as specific ablation of senescent cells using senolytic drugs was able to postpone and, in some cases, even cure them^1^. First-generation senolytics have been shown to be effective in alleviating many diseases and disabilities of aging in mouse models, and a growing number of clinical trials employing mainly the senolytics Dasatinib plus Quercetin (D+Q), or Fisetin, are underway^2,3^. However, one main limitation of current senolytics is a narrow therapeutic window, defined by the difference in EC50 between senescent and non-senescent cells, which is frequently less than one order of magnitude in concentration. This poses a significant risk for toxicity: for example, Navitoclax in concentrations typically used as senolytic (50 mg/kg) can cause thrombocytopenia^4–7^, promote pulmonary hypertension^8^, and impact bone mass in aged mice^9^. The development of more specific senolytics is still an unmet clinical need.

Senescent cells differ from their non-senescent counterparts in many respects. In addition to a stable cell cycle arrest, they display major shifts in gene expression and the production and release of a host of bioactive molecules, including cytokines, chemokines, pro-oxidative factors, and other signaling molecules, termed the senescence-associated secretory phenotype (SASP). Senescent cells are also characterized by numerous changes in their mitochondria. Mitochondrial functional capacity is best described by the ability to homeostatically maintain the mitochondrial membrane potential (MMP). The MMP is generated by proton pumping by the electron transport chain using metabolic substrates, is consumed by processes such as proton leak, adenosine triphosphate (ATP) synthesis, and transport, and is under constant homeostatic regulation. Senescent cells display low maximum respiratory capacity, increased proton leak, and low MMP at basal state^10,11^, which together indicate compromised mitochondrial functional capacity. Further accompanying changes are increased mitochondrial mass, altered mitochondrial morphology, and increased generation of reactive oxygen species (ROS)^11–15^. Together, these features have been termed senescence-associated mitochondrial dysfunction (SAMD)^16,17^. Mitochondrial functional alterations are observed during aging in isolated mitochondria from various tissues, including the liver, skeletal and cardiac muscle, and the brain. The literature shows that complex I in the electron transport chain is the predominant site of the age-dependent dysfunction, with reduced coupling and increased ROS release with complex I-linked substrates^18–22^.

Interventions that reduce mitochondrial dysfunction in senescent cells have the potential to prevent or even reverse at least some components of the senescent phenotype. Complete ablation of mitochondria in senescent cells normalized cellular ROS levels, expression of cyclin-dependent kinase inhibitors, including p16 and p21, levels of senescence-associated β-galactosidase (Sen-β-Gal), and production of multiple SASP factors to levels similar to non-senescent cells^23^. Similar suppression of the senescent phenotype was achieved by dietary restriction^24^, which improves mitochondrial function (reviewed in ref.^25^), and with dietary restriction mimetics such as rapamycin that reduce mitochondrial ROS^23^.

Conversely, mitochondrial dysfunction in senescent cells may be proposed as a targetable weakness of senescent cells. In cells with compromised mitochondrial functional capacity, mitochondria are unable to maintain MMP upon increased ATP demand or other processes that consume MMP and are more susceptible to prolonged mitochondrial permeability transition pore (mPTP) opening^26^ and possibly to (minority) mitochondrial outer membrane permeabilization (MOMP)^27,28^, both of which are linked to apoptotic cell death. Apoptosis triggered by MOMP is controlled by Bcl-2 family proteins and can be initiated in response to a plethora of intrinsic stress stimuli, such as DNA damage, oxidative stress, and endoplasmic reticulum stress. Senescent cells are typically exposed to such stresses and maintain high levels of antiapoptotic Bcl-2 family proteins^29^ to counter proapoptotic signaling and display resistance to apoptosis. BH3 mimetic drugs inhibit antiapoptotic Bcl-2 family proteins, allowing the release of apoptogenic proteins including cytochrome c as well as Smac/DIABLO from the mitochondrial intermembrane space into the cytoplasm, which drives activation of the cascade of aspartate-specific cysteine proteases (caspases) that bring about apoptotic cell death^30–32^. Suppressing the antiapoptotic action of Bcl-2 proteins with BH-3 mimetics leaves the proapoptotic signaling in senescent cells unchecked, while non-senescent cells remain unaffected, thus selectively eliminating senescent cells and acting as senolytics^33^.

Because senescent cells have a poor capacity to maintain MMP compared with non-senescent cells, we hypothesized that mild uncoupling of mitochondria can enhance the sensitivity of BH3 mimetics such as Navitoclax to induce apoptosis specifically in senescent cells. Non-senescent cells, on the other hand, would be able to tolerate mild uncoupling and thus would receive minimum effects under the same condition. We propose that the addition of low doses of mitochondrial uncoupler should enable the therapeutic efficacy of BH3 mimetics to be reached at substantially lower doses, hence broadening the therapeutic window and reducing the risk of side effects for senolytic interventions.

Here, we first characterize the mitochondrial functional capacity of senescent cells and determine the concentration range of mitochondrial uncouplers that specifically target senescent cell mitochondria. We then show that mild uncoupling enhances the senolytic activity of BH3 mimetics in vitro synergistically by up to two orders of magnitude without impacting non-senescent cells. Finally, we test the prediction that the MMP co-targeting approach enables the use of lower effective doses of Navitoclax as senolytic in a mouse model of premature aging in vivo.

## Methods

### Cell culture procedure

All cells were grown in a controlled environment of 5% CO_2_ and ambient oxygen at 37 °C in Dulbecco’s Modified Eagle’s Medium (DMEM; Sigma Aldrich, D5671), supplemented with 10% heat-inactivated fetal bovine serum (FBS; Sigma Aldrich, F9665), 100 U/ml penicillin and 100 mg/ml streptomycin (Sigma Aldrich, P4333), and 2 mM L-Glutamine (Sigma Aldrich, G7513). 1301 and HL-60 cells were cultured in RPMI 1640 with 2 mM L-Glutamine and 10% FBS.

Galactose media is prepared with 5.5 mM D-(+)-Galactose (Sigma Aldrich, G0750) replacing glucose in DMEM (no glucose, no glutamine, no phenol red) (ThermoFisher, A1443001), phenol red (Sigma Aldrich, P3532) and 10 mM 4-(2-hydroxyethyl)-1-piperazineethanesulfonic acid (HEPES) (Sigma Aldrich, H0887), 10% heat-inactivated FBS (Sigma Aldrich, F9665), 100 U/ml penicillin and 100 mg/ml streptomycin (Sigma Aldrich, P4333), and 2 mM L-Glutamine (Sigma Aldrich, G7513).

Cell culture involved human dermal fibroblasts (HDFs) derived from the foreskin of an 8-y-old male donor and neonatal HDFs sourced from neonatal foreskin (C0045C, Invitrogen). Other cell lines used included MRC5s (human fetal lung fibroblasts [ECACC 05011802]), CT-2A (mouse glioma cells [SCC194, Merck]), 1301 (Human T-cell Leukemia cells [ECACC 01051619]), and HL-60 (Acute promyelocytic leukemia [ECACC 98070106]).

Senescence was induced in fibroblasts through two mechanisms: stress-induced senescence, exposure to X-ray irradiation (20 Gy at 225 kV) 10 days prior to the experiment, and replicative senescence by culturing until the replication rate dropped below 0.2 population doublings per week. 1301 and HL-60 cells were exposed to X-ray irradiation (4 and 6 Gy, respectively, at 225 kV) 10 days prior to the experiment. CT-2A cells required 5 days of 25 μM Temozolomide (BioTechne Ltd., #2706), 2 days rest, followed by 5 days of X-ray irradiation (2 Gy at 225 kV).

### Mitochondrial function and cellular ROS

Mitochondrial respiratory capacity was examined in permeabilized cells in a Seahorse XF24 Analyzer, using a plasma membrane permeabilizer (Agilent) to permeabilize the cells (1 nM). The cells were plated on XF24 cell culture microplates and irradiated as above, and the experiments were performed at indicated time points after the irradiation using Pyruvate (10 mM), Malate (1 mM), or Succinate (4 mM) in the presence of rotenone (0.5 μM) as respiratory substrates for complex I and II, respectively. After the measurement of basal oxygen consumption rate (OCR), the following compounds were added sequentially: 4 mM adenosine diphosphate (ADP) (state 3), 1 μM oligomycin (state 4), 4 μM carbonyl cyanide-p-trifluoromethoxyphenylhydrazone (FCCP; uncoupled rate), and 2.5 μM antimycin A (complete inhibition of the electron transport chain). The respiratory control ratio (RCR) was calculated as state 3 divided by state 4 OCRs.

OCRs from intact cells were measured using Oroboros O2k. The cells grown in flasks were gently trypsinized, pelleted, resuspended in the culture media, and placed in the chamber for the experiments immediately. After the basal OCR is established, the following compounds were added sequentially: 1 μM oligomycin, FCCP in stepwise titrations (0.5 μM each to 3.5 μM), 0.5 μM rotenone, and 2.5 μM antimycin A.

Cellular and mitochondrial ROS levels were measured using 5 μM Dihydroethidium (DHE; D1168, ThermoFisher Scientific) and 0.5 μM MitoSOX^™^ (M36008, ThermoFisher Scientific), respectively, by flow cytometry. Mitochondrial mass was measured using 20 nM MitoTracker® Green FM (MTG; M7514, ThermoFisher Scientific) and MMPs using 25 nM Tetramethylrhodamine (T668, ThermoFisher Scientific) also by flow cytometry. The cells were grown on six-well plates and irradiated as above. On indicated time points, the media was replaced with fresh media containing the above probes (or vehicle control) and incubated at 37 °C for 30 min. Then the cells were gently trypsinized, resuspended with the respective media with the probes, and subjected to flow cytometry measurement immediately.

### pSLIEW and mCherry HDF generation

Early passage cells were transduced with the pSLIEW or mCherry encoding virus (whole cell localization) to generate HDF-pSLIEW (green, eGFP) and HDF-mCherry (red, mCherry). Plasmid DNA was extracted from E. coli culture under ampicillin selection using the Invitrogen PureLink HiPure Plasmid Maxiprep Kit (K210006), according to the manufacturer’s instructions. DNA concentration was determined using a nanodrop spectrophotometer (ND-1000), and plasmid DNA was stored at 1 μg/μl in 1X Tris-EDTA (TE) buffer at −20°C.

Restriction digests were performed to confirm the isolated plasmids using BAMHI, SalI, and XbaI in NEBuffer 3.1 #B7203S (New England Biolabs), PstI and HindIII in NEBuffer blue #B7002S (New England Biolabs), and EcoRI in cut smart green #B7204S (New England Biolabs), with 5% enzyme concentration, with 5% plasmid DNA. The digests were carried out at 37 °C using a thermal cycler (Veriti, ThermoFisher Scientific) for three hours and confirmed with gel electrophoresis on a 0.8% agarose gel containing 0.005% cyber green peak green DNA binder (Peolab, 37-5099).

Hek 293T packaging cells were cultured and transfected with 12 μg of equimolar plasmid DNA using the Lipofectamine 3000 reagent kit (Invitrogen, L3000001). 6% Lipofectamine 3000 in serum-free DMEM media was mixed 1:1 with diluted plasmid DNA (serum-free media with 2% plasmid DNA and 4% P3000 reagent). The generated virus from Hek 293T cells was supplemented with 6 μg/ml polybrene and added to the target cells for 12 hours at 37 °C.

### Senolysis assay

HDF-pSLIEW were plated into the interior 60 wells of black, clear-bottomed 96-well plates (Greiner Bio-one #655090) at a density of 1.5k cells per well. The 36-edge wells were filled with media. After 24 hours, the wells were supplemented with 50 μl of fresh media, and the plate was irradiated with 20 Gy of X-ray. The media was replaced after irradiation and then changed every three days for nine days postirradiation.

On day 9 postirradiation, proliferating HDFs expressing mCherry were cocultured with the irradiated senescent HDF-pSLIEW at approximately 3k cells per well. Following a 24-hour rest period, the media was changed from DMEM to Fluorobrite imaging medium (Sigma Aldrich, F9665) with 5% FBS, 100 U/ml penicillin, 100 mg/ml streptomycin, and 2 mM L-Glutamine for baseline imaging.

Images were captured in preset locations in each well using a Leica DMi-8 with an automated stage. The media was then switched to normal media containing the compound of interest, with controls receiving the same amount of solvent (dimethylsulfoxide (DMSO) or ethanol). After a set period (72 hours unless otherwise specified), the media was changed back to imaging media, and images were taken again.

Image files were manually analyzed to provide cell counts for senescent (green) and non-senescent (red) cells. Data were expressed as the percentage change between baseline and day 3 imaging, and senescent cells were normalized to their control.

### Cell viability

Cells were plated into the interior 60 wells of clear 96-well plates (Corning, 3596) and allowed to settle for 24 hours. The exterior 36 wells were filled with media. To induce senescence, cells were irradiated with 20 Gy (225 kV) and kept for 10 days, with media changes immediately after irradiation and then every 3 days. To account for proliferation, a “baseline measurement” for proliferating cells was taken by fixing a secondary plate immediately prior to administering the drugs in media to the other plates. The plate was washed with PBS, fixed with 2% paraformaldehyde (PFA) for 5 min, washed twice more, and kept at 4 °C. On the measurement day (72 hours later), the plate was taken from the fridge, allowed to warm to room temperature, and the PBS was replaced with warmed media. The plate was then processed with the remaining plates. Plates were washed with PBS and incubated in a 0.2% crystal violet/1% ethanol solution for 10 min. Plates were then washed twice by immersion in a basin of tap water and dried. Once dry, 1% SDS was added, and plates were put on a plate shaker. Absorbance was read at 590 nm. The background was subtracted using the empty exterior wells. Senescent cells were expressed as a percentage of control, while the baseline measurement was used to calculate the percentage change in cell number of the proliferating cells.

Cell viability of cells in suspension (HL-60 and 1301 leukemia cell lines) was assessed using trypan blue exclusion (Sigma Aldrich, #93595) and manually counted using a hemocytometer.

### ApoTox-Glo triplex assay

The ApoTox-Glo triplex assay (Promega, G6320) was performed in 96-well plates (Greiner Bio-one #655090) according to the manufacturer’s protocol. Results were red using a fluorescent plate reader (FLUOstar Omega, BMG), with no-cell and untreated cell controls.

### mPTP assay

mPTP opening was analyzed using the Calcein/Cobalt technique^34^. Cells were exposed to different test drugs in normal media for 3 hours, media removed, and cells counterstained with Hoechst in normal media (8 μM) for 7 min. Then, media with 10 mM CoCl_2_ was added 1:1 to wells for 7 min. Media was removed, and Calcein-AM (1 μM) was added with 5 mM CoCl_2_ for 15 min to quench fluorescence outside of the mitochondria. Media was removed and replaced with CoCl_2_ (3 μM) supplemented Fluorobrite imaging media (Sigma Aldrich, F9665; with 100 U/ml penicillin, 100 mg/ml streptomycin, and 2 mM L-Glutamine) medium for imaging using Leica DMi-8. Average intensity was recorded per cell, with an average of 400 cells per condition per repeat. The intensity following 10 μM Ionomycin was subtracted, and the results normalized to control.

### Sen-β-Gal

Cells were fixed for 5 min with 2% PFA in PBS-Mg before incubation with the staining solution as described^35^ (PBS-Mg containing 1 mg/ml X-gal, 5 mM potassium ferrocyanide, and 5 mM potassium ferricyanide, pH 6) overnight at 37 °C. Cells were co-stained with 4′,6-diamidino-2-phenylindole (DAPI) and imaged for brightfield and fluorescence (350_Ex_, 460_Em_) using a Leica DMi-8 Brightfield. Cells were quantified as positive and negative based on the presence of blue staining.

### Drug synergy analysis

Drugs and their combinations were prepared in 96-well plates in normal media with duplicate wells. The media on 96-well plates containing senescent or proliferating cells was then exchanged for drug-containing media and kept for 72 hours. Following this, crystal violet staining was performed as above, with background signal subtracted from blank exterior wells. Duplicate wells were averaged, and then values were normalized to control wells (with the same solvent concentration as drug-containing wells). Synergistic/antagonistic drug interaction was analyzed using the highest single-agent analysis with Combenefit^36^. The synergy score is expressed as the percentage of response at each combination of doses that is beyond that expected from the individual compounds.

### Animals

Male C57B1/6J mice were bought past weaning from Charles River and were maintained in groups of 3 littermates in individually ventilated cages (IVC), otherwise as described^37^. Mice were fed standard pelleted food (CRM-P formulation rodent diet, SDS diets). Cage enrichment included extra bedding and plastic domes for nesting, two cardboard tunnels per cage, rings and chains for climbing, and chewing blocks. Mice were kept on a staggered light cycle, switching to a dark cycle at 2:30 PM, with daily and weekly weighing performed under red-light conditions after that time. Mice were tunnel-handled unless otherwise required experimentally. Mice were identified by tail marking until microchipped during group allocation with the IMI-500 Read-Only Transponder (Plexx). The work was approved by the UK Home Office (PBDAFDFB0) and complied with the guiding principles for the care and use of laboratory animals. Mice were irradiated at 6–7 months of age, as previously described^38^. Mice received a 1% Baytril solution in drinking water for 2 days before and 14 days after the start and end of irradiation, respectively. Intervention for weight loss postirradiation was performed as in refs.^38^ and ^39^.

### Treatment of mice with drugs

Mice were orally gavaged with either empty vehicle, 0.5 mg/kg BW Navitoclax, or 5 mg/kg BW Navitoclax for 10 days total (5 days followed by 2 days recovery, then 5 days again). Compounds were prepared for oral gavage in 10% Polyethylene Glycol (PEG) 400; vehicle control mice were gavaged with 10% PEG400 only. Mice were simultaneously given intraperitoneal injection of either empty vehicle or 2.5 mg/kg BW BAM15 in 40% PEG400. PEG400 (8074851000) was purchased from Merck, BAM15 (Cat. No. 5737) was purchased from Biotechne, and Navitoclax (A3007-APE-100mg) was purchased from Stratech. The mice were phenotyped one month postirradiation, as below. Mice were then ranked on frailty score (low to high), average time on rotarod (high to low), and body weight (high to low). An average of each rank was then taken to get a combined ranked score. Mice were then assigned to each group according to the combined ranked scores to reduce the variability of the predosing baseline phenotypic measures between the groups.

### Mouse phenotyping

Frailty was assessed using a 30-parameter index^40^, with modifications as described^38^. Assessment was performed during the light cycle by two people who were blinded to the treatment. Scores were given independently by both assessors and then averaged. Rotarod, hanging wire, and spontaneous alternation Y-maze were performed as described^38^ during the dark cycle under red-light conditions. During the Y-maze, luminosity was 1.7lux ± 0.1 lux at the top of each arm and 0.1lux ± 0.1 at the bottom of the maze. The Y-maze duration was 8 min. For the hanging wire, previously used^38^ thick cotton bedding was substituted with 10 cm of memory foam; this provided a softer landing and eased retrieval of the mouse and cleaning.

### Liver damage assay

Liver damage was assessed using the Alanine Transaminase Activity Assay Kit (Abcam, ab105134) and the Aspartate Aminotransferase Activity Kit (Abcam, ab105135) according to the manufacturer’s instructions in a 96-well plate using a plate reader (FLUOstar Omega, BMG).

### Immunohistochemistry (IHC) and immunofluorescence (IF)

PFA-fixed paraffin-embedded tissue samples were cut from coronally embedded blocks and stained with primary/secondary antibodies as described in Table 1. IHC/IF, microscopy, and analysis were performed as otherwise described^39,41^. Microscopy for IF and IHC was performed using a DM18 fluorescence microscope (Leica, Germany). Microglia density and soma size were measured described^39,41^ to give ibal + cells per mm^2^ and soma size (μm^2^). Doublecortin- (DCX-) positive cells were counted and divided by the perimeter of the lateral ventricle to give DCX^+^ cells per mm. Dentate Gyrus width was measured every 120 μm along the blades; an average of these measurements was then taken for each mouse. Images were analyzed using the Fiji distribution of ImageJ2.

### Navitoclax literature search

To survey the current dosing regimens using Navitoclax in the context of aging and as an anti-senescence therapy, a literature search on PubMed was performed as ([Navitoclax] OR [ABT-263]) AND (mouse). Articles ascertaining to senescence, senolytics, and aging in mice were selected from this list for inclusion (last updated 4 April 2023). Route of administration (oral gavage, intraperitoneal, intramuscular, intravenous, intradermal, and topical administration routes were identified), schedule of dosing (including total days), and doses used each time were manually extracted from each article and tabled. Where these data were not clear from the article, the authors were approached. If these data could not be obtained, the articles were excluded from the results. As we were providing Navitoclax by oral gavage, articles using this route of administration were selected, and the total duration of administration and dose per dosing were tabled as a.csv. A kernel density estimation plot was created using Seaborn 0.11.1.

### Statistics

Data were collated in Microsoft Excel, with all statistics and graphing performed in GraphPad Prism 9. One-way and two-way analysis of variance (ANOVA) and nonlinear regression analysis tests were performed using GraphPad Prism 9. Drug synergy was performed as described above.

The in vivo study was designed to evaluate if a lower (than previously administered by us) dose of Navitoclax (0.5 mg/kg) would have an effect if combined with the mitochondrial uncoupler BAM15 (2.5 mg/kg). Low Navitoclax and BAM15, alone and in combination, were compared to an untreated control group (vehicle-only gavage and I.P.). This was set up as a two-way ANOVA and analyzed in GraphPad Prism 9.

In order to assess if low Navitoclax and BAM15 in combination would be comparable to our previously used higher dose of Navitoclax (5 mg/kg), the groups control, Nav (5 mg/kg), and Nav (0.5 mg/kg)/BAM15 (2.5 mg/kg) were compared separately with a one-way ANOVA. To avoid repeating comparisons with the two-way ANOVA, the Nav/BAM15 group was not compared to the control in post hoc for this test.

## Results

### SAMD

**Figure 1:**
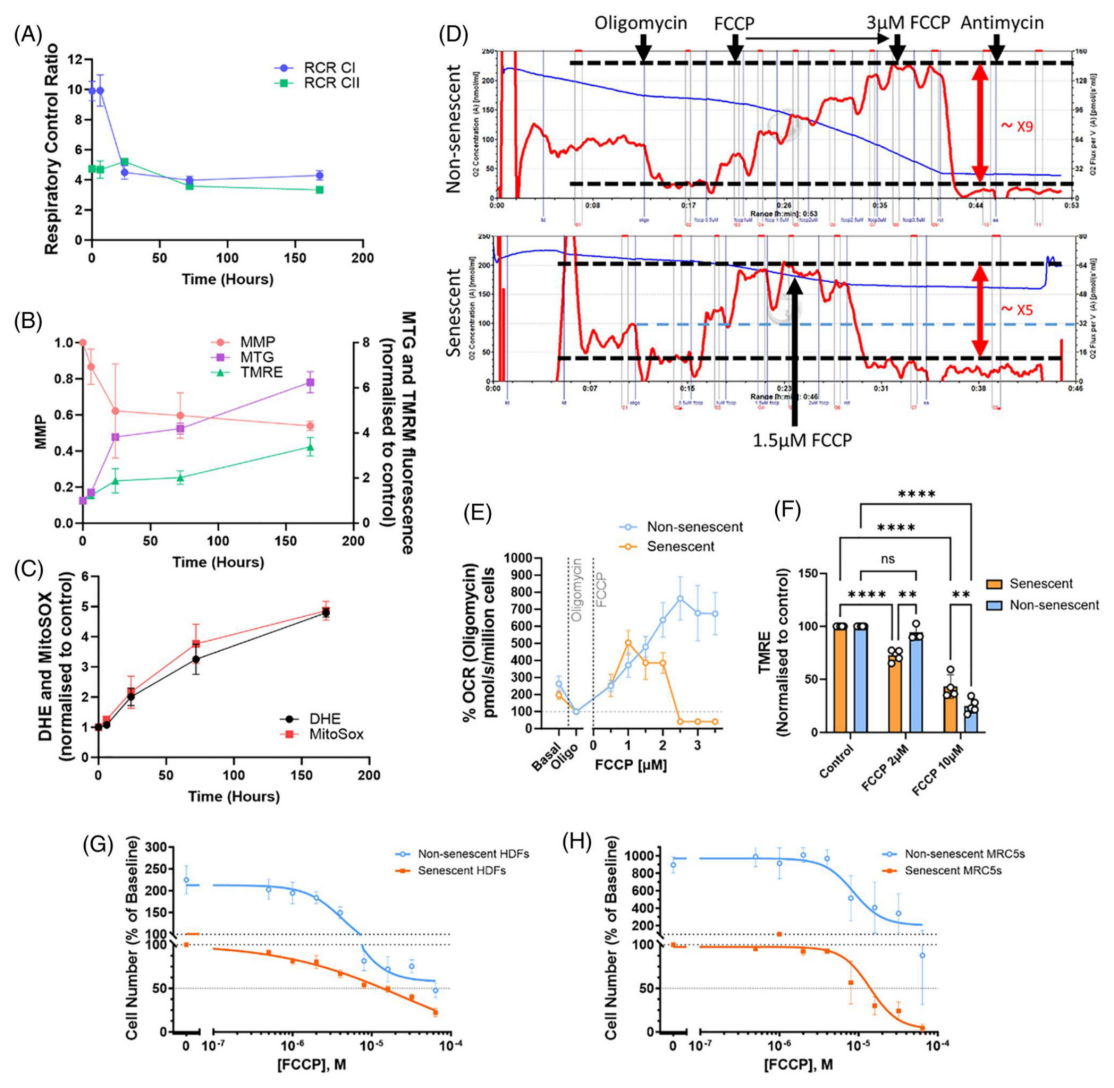
Mitochondrial functional changes during senescence development. **(A–C)** Senescence was induced in human dermal fibroblasts (HDFs) by 20 Gy irradiation at time 0, and the development of mitochondrial dysfunction was measured over the following 7 days. Time 0 indicates pre-irradiation. **(A)** Respiratory control ratios with complex I (blue) and complex II (green)-linked substrates. **(B)** Mitochondrial mass (measured as MitoTracker^®^ Green FM fluorescence intensity, purple), Tetramethylrhodamine (TMRM) fluorescence intensity (green), and mitochondrial membrane potential (red). **(C)** Reactive oxygen species production measured as Dihydroethidium (black) and MitoSOX™ (red) fluorescence. **(D)** Representative traces of intact cell oxygen consumption rates (OCRS; red lines) measured in Oroboros O2k, with sequential additions of indicated compounds. Carbonyl cyanide-p-trifluoromethoxyphenylhydrazone (FCCP) was titrated by 0.5 μM at each addition. Non-senescent cells can increase OCR about ninefold compared to the resting state (in the presence of Oligomycin), whereas this is decreased to fivefold in senescent cells. **(E)** Summary of 6 (non-senescent) and 8 (senescent) independent Oroboros experiments. Values were obtained at steady-state after each addition of compounds and presented as % of OCR at the resting state (in the presence of oligomycin). **(F)** Changes in TMRM in senescent (red) and non-senescent (blue) HDFs under the indicated concentrations of FCCP relative to the control condition (no uncoupler). Data are mean ± standard error of the mean (SEM), N=3–8, *p < 0.05, **p ≤ 0.005, ****p ≤ 0.0005, ****p ≤ 0.0001. **(G**,**H)** Change in number of senescent (red) and non-senescent (blue) HDFs (panel (G)) and MRC5 cells (panel (H)) after 3 days of treatment with the indicated FCCP concentrations. Data are mean ± SEM, N = 3.

To understand the development of senescence-associated functional changes in mitochondria, we performed a concerted timecourse analysis of mitochondrial respiratory capacity, membrane potential, and ROS levels following the induction of stress-induced senescence (Fig. 1A–C). Human fibroblasts were treated with 20 Gy irradiation, which is sufficient to induce senescence in 100% of the cells^11,12^. Mitochondrial OCR using either complex I (Pyruvate and Malate) or II (Succinate)-linked substrates was examined in permeabilized cells to determine mitochondrial-coupled respiration. The RCR with complex I-linked substrate was retained for at least 6 hours after irradiation but dropped by about 50% within the next 18 hours and then remained at low levels during further maturation of the senescent phenotype (Fig. 1A), which was accompanied by a similar change in MMP (Fig. 1B). There was also a simultaneous increase in mitochondrial mass per cell, as measured by MTG fluorescence (Fig. 1B). On the other hand, complex II-linked RCR remained largely unaffected (Fig. 1A). ROS levels in both mitochondria (MitoSOX™) and cytoplasm (DHE) only started to increase after complete reduction of complex I-linked RCR and MMP at day 1 and continued to do so over at least one week (Fig. 1C). Thus, in senescent cells, decreased coupled respiration at complex I is a key functional change and an early event preceding the increase of ROS.

To further establish the link between low mitochondrial functional capacity and the ability to maintain MMP in senescent cells and to explore the utility of mild uncoupling as a specific target for senescent cells, cellular OCR was measured with titrations of the mitochondrial uncoupler FCCP by 0.5−3.5 μM in both senescent and non-senescent cells. As shown before by us and others^10,12^, “spare respiratory capacity” (max OCR-basal OCR) was higher in non-senescent cells than in senescent cells; non-senescent cells tolerate uncoupling to more than 3 μM, resulting in about eightfold higher maximum OCR compared with the resting state (in the presence of oligomycin), but respiration in the senescent cells collapses at an FCCP concentration beyond 2 μM, achieving only about fivefold higher maximum OCR compared to the resting state (Fig. 1D,E). 2 μM FCCP was sufficient to reduce the MMP of senescent human fibroblasts but not that of non-senescent fibroblasts. As expected, 10 μM FCCP reduced MMP in both senescent and non-senescent cells (Fig. 1F). However, the uncoupler FCCP on its own in increasing concentrations did not lead to a sufficiently differential reduction in cell viability between senescent and non-senescent cells. (Fig. 1G,H).

### Mitochondrial uncouplers synergistically increase the senolytic efficacy of BH3 mimetics

Having established the level of mild uncoupling that specifically targets MMP in senescent cells, we tested for a synergistic interaction between mild uncoupling and BH3 mimetics as senolytics.

**Figure 2:**
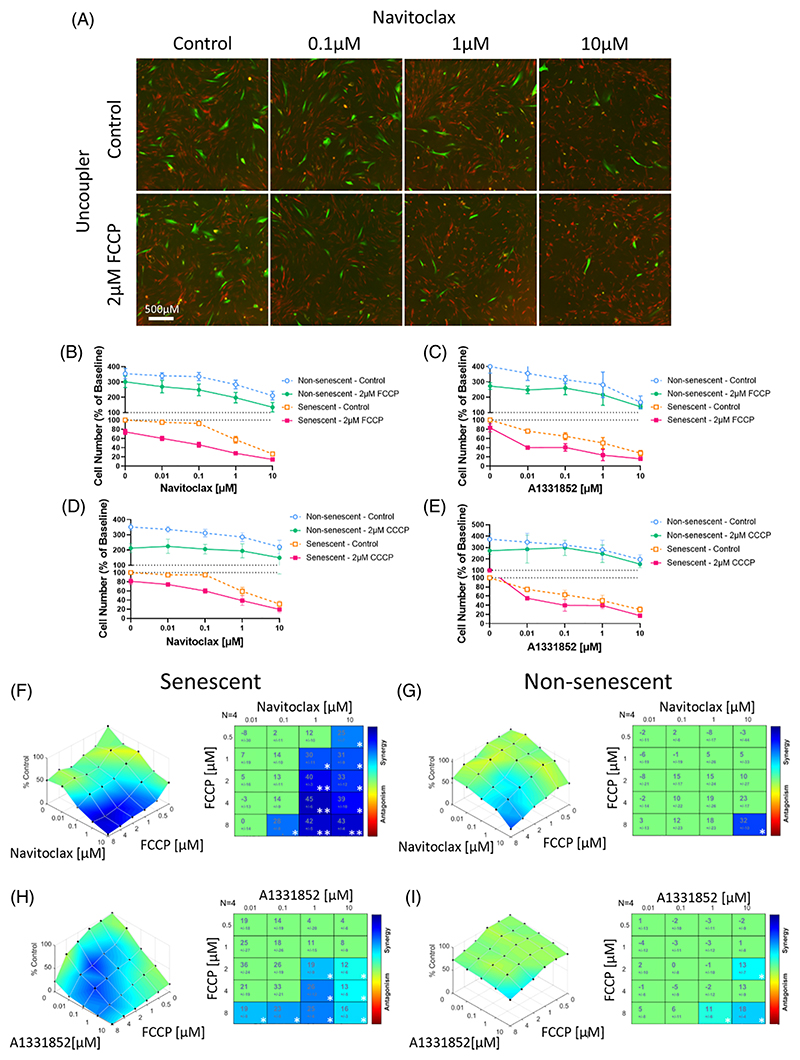
BH3 mimetics and uncouplers synergize in killing specifically senescent cells. **(A)** Fluorescence micrographs of HDFs in X-ray irradiation-induced senescence (green) cocultured with unirradiated non-senescent HDFs (red) for three days in the presence of increasing doses of Navitoclax with or without 2 μM of FCCP. **(B–E)** Titration curves of senescent (red/yellow) and non-senescent (green/blue) HDFs with increasing concentrations of BH3 mimetics (panels (B) and (D)) Navitoclax and (panels (C) and (E)) A1331852, with or without (panels (B) and (C)) 2 μM FCCP or (panels (D) and (E)) CCCP. Data are mean ± SEM, N = 3–6 independent experiments. **(F–I)** The Highest Single Agent analysis showing cell viability compared to control with combinations of BH3 mimetic and FCCP in senescent (left) and non-senescent cells (right). Synergy/antagonism is shown by color and synergy score with change from expected value from either single compound for the (panels (F) and (G)) Navitoclax/FCCP and (panels (H) and (I)) A1331852/FCCP combinations on (panels (F) and (H)) senescent and (panels (G) and (I)) non-senescent HDFs. N = 4, significance shown by *p ≤ 0.05, **p ≤ 0.005.

We treated senescent and non-senescent HDFS in coculture with increasing concentrations of Navitoclax, which inhibits both Bcl-2 and Bcl-xL^42^, with or without the mitochondrial uncoupler FCCP at 2 μM and measured the change in cell number after 72 hours of treatment (Fig. 2A). By coculturing HDFs constitutively expressing pSLIEW (containing EGFP) in stress-induced senescence by irradiation (IR) with proliferating cells expressing mCherry and tracking changes in cell number by imaging, this model allowed us to take any interactions between senescent and non-senescent cells under the senolytics into account. The senescent state of HDFs at 10 days past 20 Gy irradiation had been established before and was confirmed here (Suppl. Fig. S2·1). In accordance with data from Figure 1, 2 μM FCCP had only a small effect on the proliferation of non-senescent HDF during the 72 hours. However, the numbers of surviving senescent HDFs were lower under all Navitoclax concentrations when combined with 2 μM FCCP (Fig. 2A). Importantly, the combination with 2 μM FCCP enhanced the EC50 of Navitoclax in senescent cells by about two orders of magnitude, whereas it had a much smaller effect on non-senescent HDFs (Fig. 2B). When non-senescent cells were rendered quiescent by contact inhibition, they became even more resistant (data not shown). Similar results were obtained for combinations of multiple mitochondrial uncouplers and BH3 mimetics: Navitoclax and CCCP (Fig. 2D), or the specific Bcl-xL inhibitor A1331852 and either FCCP (Fig. 2C) or CCCP (Fig. 2E).

To test for synergy between BH3-mimetics and mitochondrial uncouplers, we performed titration experiments using combinations of either Navitoclax or A1331852 and FCCP over a range of concentrations each. The highest single agent analysis using Combenefit showed strong synergism in senescent HDFs (Fig. 2F,H), but there was no significant interaction in non-senescent HDFs until the highest tested doses of both compounds (Fig. 2G,I).

To assess the mechanism of senolytic function of the combination of BH3-mimetics and mitochondrial uncouplers, cell viability, cytotoxicity, and caspase3/7 activity were examined (Promega ApoTox-Glo Triplex Assay System) in senescent and non-senescent HDFs after 24 hours of treatment with Navitoclax alone (Suppl. Fig. S2-2a) or combined with 2 μM FCCP (Suppl. Fig. S2-2b). There was very little induction of caspase activity in non-senescent cells; however, the induction of caspase activity by Navitoclax in senescent HDFs was potentiated by its combination with FCCP (Suppl. Fig. S2-2a,b). Although a low concentration of Navitoclax (0.1 μM) enhanced caspase activity in both non-senescent and senescent cells, a significant interaction between Navitoclax and FCCP was only found in senescent cells (not shown). Readouts for cell viability and cytotoxicity were independent of increasing Navitoclax concentrations with or without FCCP (Suppl. Fig. S2-2a,b), and the addition of the pan-caspase inhibitor z-VAD-FMK for 24 hours reduced senescent cell loss under Navitoclax alone or with FCCP (Suppl. Fig. S2-2c). Thus, these data together indicate that the combination of Navitoclax and FCCP induces apoptotic but not necrotic cell death. The combination treatment triggered a significant opening of the mPTP after 3 hours of treatment, which was rescued by cyclosporin-A, an inhibitor of mPTP opening (Suppl. Fig. S2.2d,e).

Navitoclax in combination with FCCP and CCCP also showed higher senolytic efficacy than Navitoclax alone in replicatively senescent HDFs (Suppl. Fig. S2-3a,b) and in irradiation and replicatively induced MRC5 embryonic lung fibroblasts (Suppl. Fig. S2.3c,d).

To test the specificity of the combination of BH3 mimetics and mitochondrial uncouplers as senolytics, we first tested an uncoupler that does not selectively target the MMP. 2,4-Dinitrophenol (DNP) is a weak uncoupler of both mitochondrial and other cellular membranes and prevents the uptake of inorganic phosphates into the mitochondria, leading to the accumulation of potassium and phosphate 43-45. DNP concentrations up to 40 μM had only mild effects on the senolytic activity of Navitoclax (Suppl. Fig. S2-4a) or A1331852 (Suppl. Fig. S2-4b) in HDFs. To confirm that a reduction in MMP, rather than increasing cellular energetic output, enhances the senolytic function of BH3 mimetics, Monensin was used in combination with Navitoclax. Monensin is an ionophore which selectively transports sodium cations across lipid membranes and increases cellular energy expenditure via both glycolytic and oxidative phosphorylation. Monensin synergistically increased the killing effect of Navitoclax in both senescent and non-senescent cells equally, negating any specificity for senescent cells (Suppl. Fig. S2-4c,d).

We next combined mitochondrial uncouplers with senolytics that target other antiapoptotic pathways that are upregulated in senescent cells. The cardiac glycoside Digoxin reduces pH by inhibiting the Na^+^/K^+^-ATPase pump, depolarizing the plasma membrane, and causing a disbalanced electrochemical gradient within the cell, to which senescent cells appear to be more susceptible^46^. In HDFs, however, the therapeutic window between senescent and non-senescent cells for Digoxin was small and was not improved by the addition of either CCCP or FCCP (Suppl. Fig. S2-5a,b). D+Q inhibits multiple tyrosine kinases, PI3K, and serpines and has been identified as senolytic in fat and endothelial cells, but with limited senolytic activity in human fibroblasts^47^. While uncouplers FCCP and CCCP reduced the numbers of surviving senescent cells in some experiments, this was independent of an interaction with Dasatinib alone (Suppl. Fig. S2-5c,d) or D+Q in combination (Suppl. Fig. S2-5e,f). The naturally occurring flavonoid Fisetin was reported to have mild senolytic activity in fibroblasts^48,49^. We did not see significant senolytic activity of Fisetin in our assay, and this was not improved by combination with FCCP (Suppl. Fig. S2.5g). We conclude that only combinations of BH3 mimetics with mitochondrial uncouplers synergistically enhance senolytic sensitivity and specificity in fibroblasts.

It had been shown previously that chronic exposure to FCCP can have negative effects in human fibroblasts, including the induction of premature senescence^50^. BAM15 is a recently discovered mitochondrial protonophore with a longer-lasting effect on respiratory kinetics and with improved safety profile that has been demonstrated in vitro and in vivo^51–53^.

**Figure 3:**
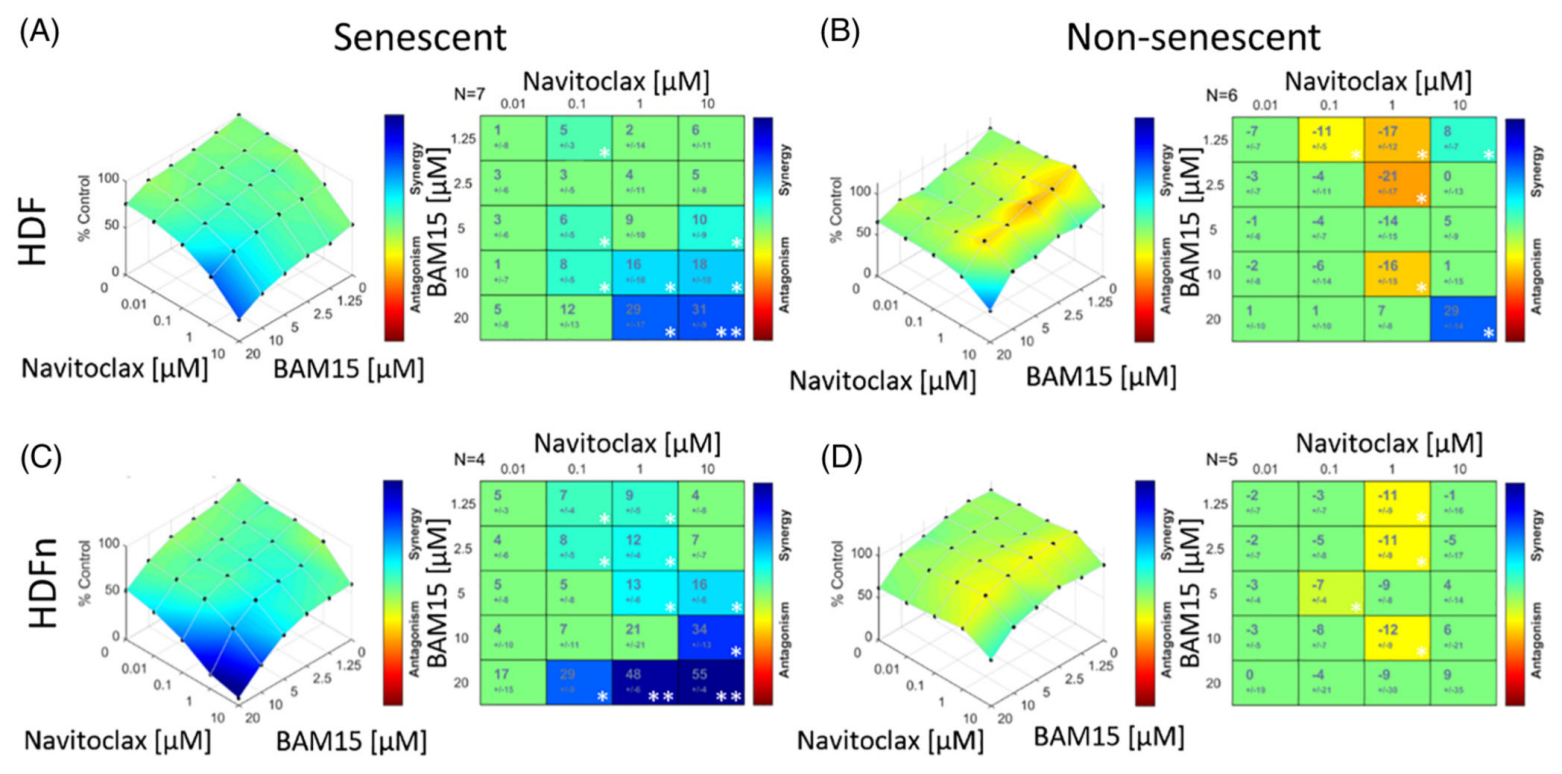
Combination of Navitoclax and BAM15 is synergistic for senolytic activity. The Highest Single Agent analysis showing cell viability compared to control with combinations of BH3 mimetic and BAM15 in senescent (left) and non-senescent cells (right). Synergy/antagonism is shown by color and synergy score with change from expected value from either single compound for senescent and non-senescent **(A**,**B)** adult HDFs and **(C**,**D)** neonatal HDFs for BAM15 and Navitoclax, N=4–6. The significance shown by *p ≤0.05 and **p ≤ 0.005.

Although chronic exposure to 2 μM FCCP over 10 days reduced the growth rate of HDFS (Suppl. Fig. S3-1a) and induced senescence, as shown by increased fractions of cells displaying karyomegaly (Suppl. Fig. S3-1b) and expressing Sen-β-Gal (Suppl. Fig. S3-1c,d), 10 μM BAM15 did not. When tested for its effect on cell viability on its own, the 10 μM range of BAM15 could remove a fraction of senescent cells. (Suppl. Fig. S3-2a–f). Overall, BAM15 had a slightly higher EC50 for fibroblasts than FCCP (Suppl. Fig. S3-2a-f, compare with Fig. 1), but only a narrow difference between senescent and non-senescent fibroblasts (Suppl. Fig. S3-2a,d). Navitoclax combined with BAM15 killed stress-induced and replicatively senescent, but not non-senescent HDFs synergistically, from adult (Fig. 3A,B; Suppl. Fig. S3-3a,b) and neonatal donors (Fig. 3C,D), as well as MRC5 cells (Suppl. Fig. S3c,d). Replicatively senescent cells showed particular vulnerability to the combination (Suppl. Fig. S3-3b,d), as did chemotherapy-induced senescent MRC5 cells (Suppl. Fig. S3-3e).

10 μM BAM15 enhanced Navitoclax-induced caspase activation in senescent but not non-senescent fibroblasts, and it did not induce necrosis (Suppl. Fig. S3-4a). Furthermore, three hours of combination treatment with Navitoclax and BAM15 induced more mPTP opening than Navitoclax alone, which was rescued by coadministration of cyclosporin-A (Suppl. Fig. S3-4b,c).

Cultured cells in nutrient—rich and high-glucose—media can readily rely on glycolysis when mitochondrial ATP generation is compromised, and a typical cell culture environment can fail to recapitulate the in vivo environment where mitochondria generate almost all the energy in the form of ATP. The use of galactose as a replacement to glucose in culture media, which enhances mitochondria-driven ATP generation over glycolysis, often better reveals the in vivo scenario of drug toxicities around mitochondria^54^. When tested in galactose media, the concentration of the compounds required for a synergistic effect in stress-induced senescent HDFs was reduced (Suppl. Fig. S3-5).

Taken together, our data show that the mitochondrial uncoupler BAM15 also synergistically enhances the senolytic activity of Navitoclax in human fibroblasts.

It is widely hypothesized that therapy-induced senescence of tumor and niche cells can be a significant cause of insufficient tumor therapy response and that this might be improved by adjuvant senolytic intervention past radio/chemotherapy^55–59^. Tumor cells frequently show evidence of mitochondrial dysfunction, even without senescence induction. We therefore tested the efficacy of the combination of mitochondrial uncoupler and Navitoclax on the human leukemia cell lines 1301 and HL60 and the mouse glioblastoma line CT-2A. BAM15 alone was effective at arresting the growth of proliferating and therapy-treated leukemia and glioblastoma cells (Suppl. Fig. S3-6a–d) and potentiated the effect of Navitoclax against both replicating and therapy-treated cancer cells (Suppl. Fig. S3-6a–c) with a synergistic interaction (Suppl. Fig. S3-6d). Senescent cancer cells can show resistance to Bcl-xL inhibition through upregulation of another antiapoptotic Bcl-2 family member, Mcl-1^60,61^. Using the Mcl-1 inhibitor (S63845) potentiated the effect of Navitoclax against therapy-treated CT-2A cells and worked in combination with BAM15 to further increase cell killing by another order of magnitude (Suppl. Fig. S3-7a). This effect could also be observed in stress-induced senescent MRC5 cells (Suppl. Fig. S3-7b). However, Mcl-1 inhibitors have dose-limiting toxicities that could limit their use, and we found that at higher doses, the combination of Navitoclax and S63845 was toxic to non-senescent fibroblasts (Suppl. Fig. S3-7c).

### In combination with the mitochondrial uncoupler BAM15, very low concentrations of Navitoclax are sufficient to rescue premature aging in mice

**Figure 4:**
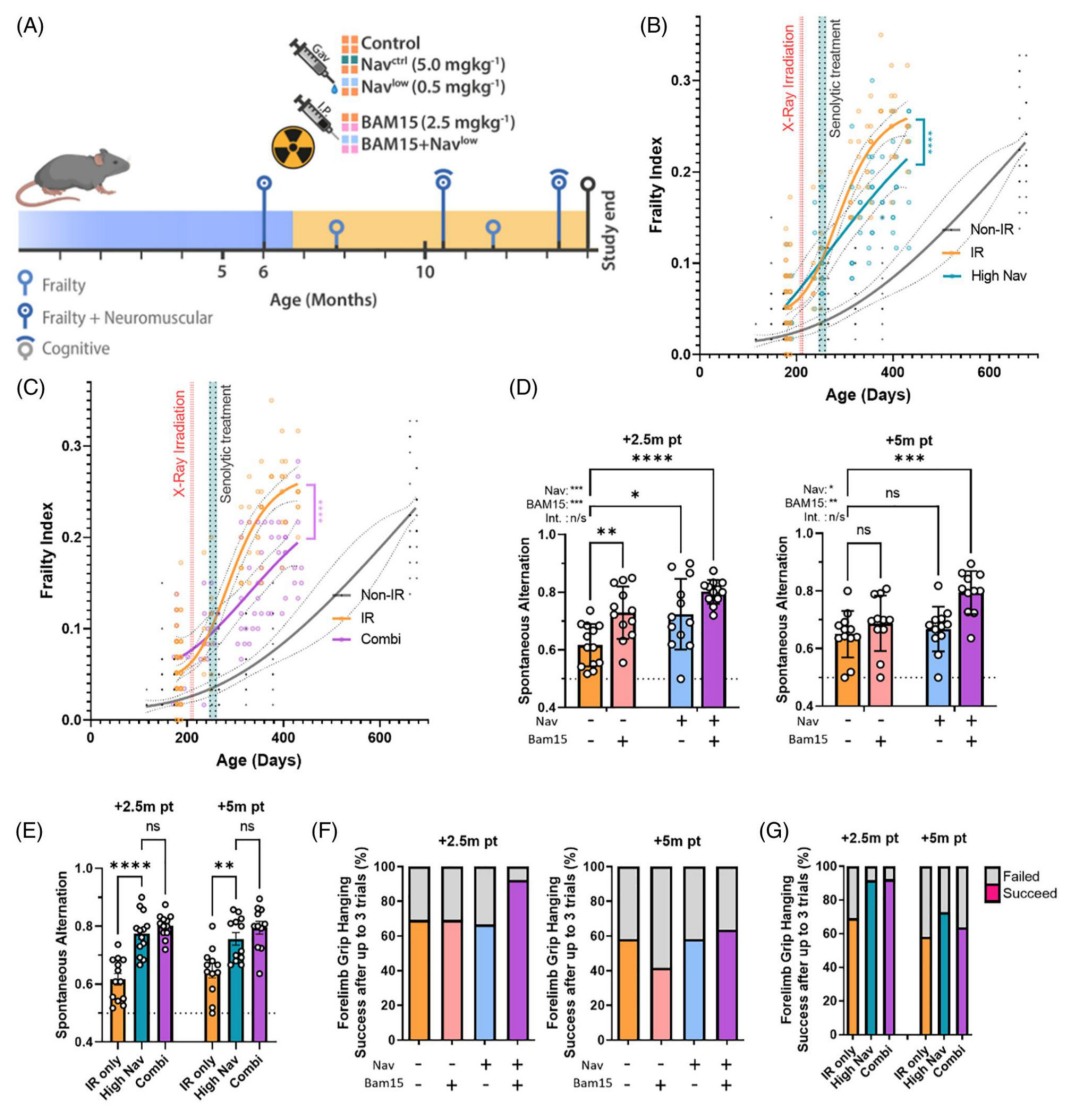
In combination with BAM15, a low dose of Navitoclax effectively rescued accelerated frailty and memory loss in irradiated mice. **(A)** Experimental design. Mice were irradiated at six months of age and treated one month later with either a vehicle, a low or high dose of Navitoclax (0.5 and 5 mg/kg, respectively), BAM15 (2.5 mg/kg), or a combination of low-dose Navitoclax and BAM15. Mice were longitudinally phenotyped for frailty, cognition, and neuromuscular performance over a further seven months. **(B)** Frailty index (FI) versus mouse age for nonirradiated (non-IR, black), irradiated (IR, orange), and irradiated plus treated with high Navitoclax (blue) mice. **(C)** FI versus mouse age for nonirradiated (non-IR, black), irradiated (IR, orange), and irradiated plus treated with the combination of low Navitoclax plus BAM15 (pink) mice. In panels (B) and (C), irradiation and treatment times are indicated by vertical lines. Dots indicate FI for individual mice; regression lines and 95% confidence intervals are indicated by bold and dotted lines, respectively; and a variable slope (four points) nonlinear regression analysis is shown. Nonirradiated data are reproduced from Fielder et al. (2019)^38^ for comparison. **(D)** Short-term memory as assessed by spontaneous alternation in a Y-maze in irradiated mice treated with either sham, BAM15, low Navitoclax, or the combination of both at 2.5 months (left) and 5 months (right) past treatment. Comparisons by two-way analysis of variance (ANOVA) with Šídák’s multiple comparisons post hoc test against the untreated irradiated control: *p ≤ 0.05, **p ≤ 0.005, ***p ≤ 0.0005, ****p < 0.0001. Data are mean ± SEM, N ≥ 11. (**E**) Short-term memory as assessed by spontaneous alternation in a Y-maze in irradiated mice treated with either sham, high Navitoclax, or the combination of BAM15 and low Navitoclax at 2.5 months (left) and 5 months (right) past treatment. Data are mean ± SEM, N ≥ 11. Comparisons by one-way ANOVA with Šídák’s multiple comparisons post hoc test against the untreated irradiated control: *p ≤ 0.05, **p ≤0.005, ***p ≤ 0.0005, ****p < 0.0001. (**F**) Success rate in the Hanging Wire test for irradiated mice treated with either sham, BAM15, low Navitoclax, or the combination of both at 2.5 (left) and 5 (right) months past treatment. N ≥ 11. (**G**) Success rate in the Hanging Wire test for irradiated mice treated with either sham, high Navitoclax, or the combination of BAM15 and low Navitoclax at 2.5 months (left) and 5 months (right) past treatment. N ≥ 11.

Navitoclax is well established as an effective senolytic drug capable of alleviating multiple aging- and/or stress-associated degenerative conditions if given in concentrations between 5 and 50 mg/kg BW to mice (Suppl. Fig. S4-1)^39,62–70^. In a model of irradiation-induced premature aging^38^, we established previously that male C57Bl/6J mice that received fractionated whole-body irradiation at an age of about six months developed frailty at about twice the normal aging rate and showed decreased neuromuscular and cognitive function all of which were rescued by a short course of 5 mg/kg BW Navitoclax at one month after irradiation^39^. Here, we employed the same model to test the efficacy of the combination of low Navitoclax with BAM15 in vivo. In addition to the previous dose of Navitoclax at 5 mg/kg/day as in ref.^39^, we now treated the mice with either a tenfold lower dose of Navitoclax at 0.5 mg/kg/day, BAM15 at 2.5 mg/kg/day, or a combination of low Navitoclax at 0.5 mg/kg/day and BAM15 at 2.5 mg/kg/day (Fig. 4A). Treatment was for two rounds of five days, separated by two days. This design allowed us to address two questions in parallel, namely, whether the combination was better than solo treatments alone and how it compared to an intervention with the previously used higher Navitoclax doses.

None of the short-term treatments had a lasting effect on body weight (Suppl. Fig. S4-2a). Irradiated mice showed an early increase in frailty after irradiation, and this was ameliorated by high Navitoclax (5 mg/kg; Fig. 4B), as found before^38,39^. A low dose of Navitoclax or BAM15 alone resulted only in minor reductions of frailty progression (Suppl. Fig. S4-2b,c). However, the combination of BAM15 with a low Navitoclax dose was as effective in reducing frailty as a tenfold higher dose of Navitoclax alone (Fig. 4C, compare to Fig. 4B).

Low Navitoclax, BAM15, and their combination improved cognitive function when assessed by measuring spontaneous alternation in a Y-maze at 2.5 months after treatment (Fig. 4D) in a two-way ANOVA analysis. In a post hoc analysis, only the combination treatment remained significantly different from controls at a later time point, that is, five months after treatment (Fig. 4D). The higher dose of Navitoclax at 5 mg/kg/day also significantly improved cognitive performance at both time points, to the same degree as the combination intervention (Fig. 4E).

Assessment of neuromuscular coordination by hanging wire test showed initial improvements by the combination treatment over low Navitoclax or BAM15 alone, although the effects were lost at the late time point (Fig. 4F). An equal degree of improvement over irradiated controls was seen between the higher Navitoclax and the combination treatment, but only for the earlier time point (Fig. 4G). Physical endurance, at either early or late time points after treatment, as measured by accelerating rotarod, was not changed by any treatment (Suppl. Fig. S4-2d,f), and neither was liver damage, as assessed by the activity of alanine transaminase (ALT) and aspartate aminotransferase (AST) in serum (Suppl. Fig. 4-2e,g).

Given the persistent improvements in memory by the combination treatment (Fig. 4D,E), we examined markers of neuroinflammation in the hippocampus.

**Figure 5:**
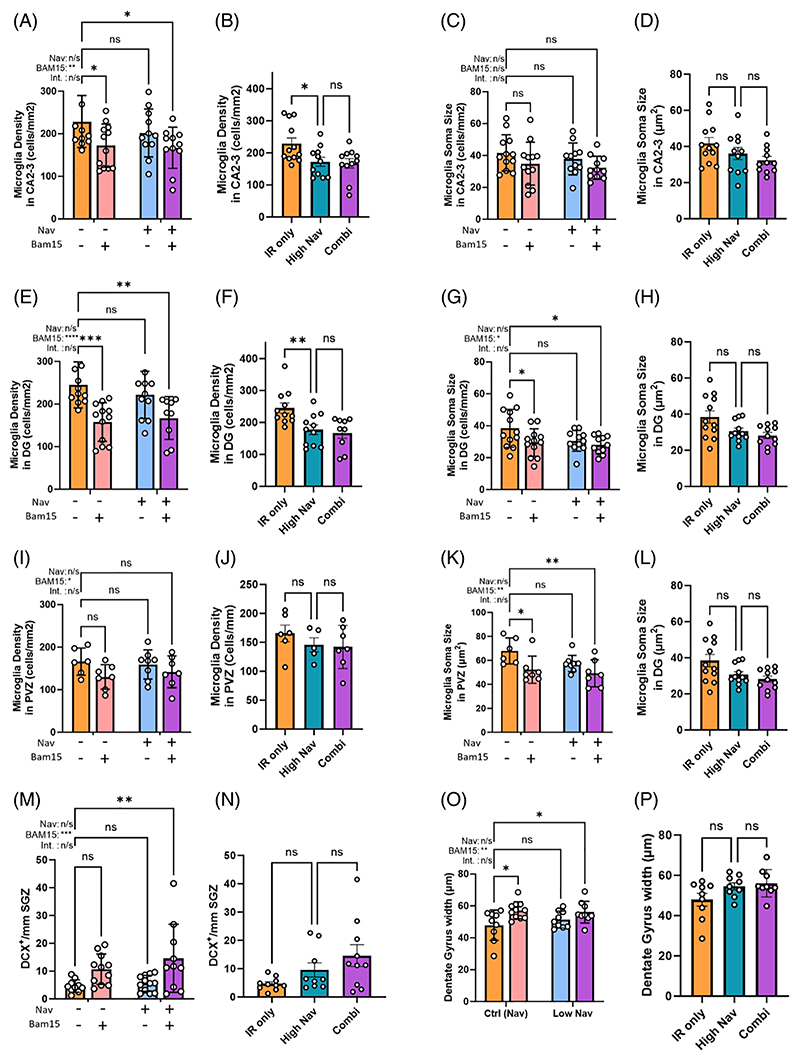
In combination with BAM15, a low dose of Navitoclax effectively reduced neuroinflammation at seven months past treatment. Microglia density and soma size in the **(A–D)** CA2-3 region of the hippocampus, **(E–H)** the dentate gyrus, and the **(I–L)** subventricular zone in irradiated mice treated with either sham, BAM15, low Navitoclax, or the combination. **(M**,**N)** The numbers of doublecortin-positive cells along the subventricular zone. **(O**,**P)** Thickness of the Dentate Gyrus. Data are mean ± SEM, sections from N = 5–12 animals. Two-way ANOVA with Šídák’s multiple comparisons post hoc for Control, BAM15, Low Nav, and Combination. Comparisons by one-way ANOVA with Šídák’s multiple comparisons post hoc test against the untreated irradiated control for Control/High Nav/Combination, *p ≤ 0.05, **p ≤ 0.005.

We measured the density of microglia (Fig. 5A,B) and their soma size (Fig. 5C,D) as a proxy of microglia activation in the CA2-3 region of the hippocampus, the dentate gyrus (Fig. 5E–H), and the periventricular zone (Fig. 5I–L) and found reductions of both parameters after the combination treatment that were significant in most cases. A two-way ANOVA indicated significant effects for BAM15 on microglia density (all examined regions) and for microglia soma size (dentate gyrus and periventricular zone). Reductions in both parameters by the combination of a low dose of Navitoclax and BAM15 were not significantly different than those by the higher dose of Navitoclax alone (Fig. 5B,D, F,H,J,L). In accordance with a reduction in neuroinflammation markers, we also observed enhanced density of DCX-positive neurons in the subventricular zone as a proxy indicator of improved neurogenesis (Fig. 5M,N), which was significant in a post hoc analysis for the combination treatment. Again, two-way ANOVA analysis indicated a significant effect for BAM15 (Fig. 5M), and low Navitoclax together with BAM15 tended to be even more effective than the higher Navitoclax (Fig. 5N). While DCX-positive cells were not common in the dentate gyrus, BAM15 showed a significant effect on dentate gyrus width, and post hoc tests showed a significant change both alone and in combination with Navitoclax (Fig. 5O), which was not significantly different from the higher dose of Navitoclax (Fig. 5P). Neither high nor low Navitoclax interventions (with or without BAM15) changed LaminB1 expression or neuron nuclear size as indicators of senescence in the hippocampus (not shown), suggesting that the effects of the senolytic interventions on neuroinflammation and cognitive function might not necessarily be driven by directly reducing hippocampal neuron senescence. Together, our data show that a short intervention with a combination of a low dose of Navitoclax and BAM15 is sufficient to reduce late premature frailty, persistent neuroinflammation, and cognitive impairment caused by sublethal irradiation as much as a high dose of Navitoclax.

## Discussion

Our kinetic study of mitochondrial functional changes during damage-induced senescence in vitro showed decreased complex I-dependent coupled respiration and decreased MMP as the earliest and persistent events preceding the increase in ROS production. Complex I-dependent changes typically characterize mitochondrial functional changes in aged tissues, suggesting a mechanistic similarity between cell senescence and tissue aging. Our study provides supportive evidence that mitochondrial dysfunction observed in senescent cells in vitro, with a limited ability to maintain MMP in response to mild uncoupling, is also functionally relevant in vivo: the mitochondrial uncoupler BAM15 was able to synergize with Navitoclax such that the same beneficial effects as 10 times higher dose were achieved in a mouse model of premature aging.

The experiments on cells in culture were performed in an artificial condition, for example, in nutrient-rich media with high glucose. Under such conditions, senescent cells can compensate for their compromised mitochondrial functional capacity by shifting toward a more glycolytic energy metabolism. However, in vivo, where oxidative metabolism is more dominant, it can be expected that the consequences of mitochondrial dysfunction in senescent cells could be even more serious. This could be partially mimicked by replacing glucose in media with galactose, which we found reduced the required concentrations for synergy of BAM15 and Navitoclax against senescent cells.

An important implication of the existence of populations of cells harboring mitochondria with a low ability to maintain MMP in vivo should be noted. Drug conjugates with a triphenylphosphonium (TPP^+^) lipophilic cation are frequently used in mitochondrial targeting approaches^71–73^ because they enable rapid, several-hundred-fold accumulation of the conjugate into mitochondria in response to MMP^71^. Importantly, such strategies typically aim to target “diseased” cells, where mitochondria frequently exhibit compromised functional capacity and a correspondingly lower MMP. Examples include approaches to target cancer and senescent cells with cytotoxic drugs (Mito-Tamoxifen) or cancer cells with Mito-metformin^74–76^. However, because of the low MMP in senescent or cancer cells, TPP^+^-conjugated drugs might preferentially concentrate in normal mitochondria in healthy cells in vivo rather than in senescent or cancer cells, which can lead to unexpected outcomes. Conversely, TPP^+^-conjugated drugs may be more productive if mitochondria in non-senescent (or noncancerous) cells are targeted, for example, in order to protect them against adjuvant cytotoxic interventions. For example, mitochondria-targeted antioxidants (MitoVit-E and MitoQ) have been shown to bring protective effects against oxidative stress-induced damage, such as telomere shortening^77,78^.

There was a clear synergism between mitochondrial uncouplers and BH3 mimetics in senescent but not non-senescent fibroblasts, with the combination having up to 100-fold higher specific senolytic activity. This was achieved specifically by mitochondrial uncouplers and not by other drugs that cause generic upregulation of OCRs and metabolic outputs. Furthermore, there was no senescence-specific synergistic activity in combinations of mitochondrial uncouplers with senolytics that do not directly target antiapoptotic Bcl-2 family proteins. Our data suggested that the inability of senescent cells to maintain MMP under mild uncoupling significantly enhances their sensitivity to BH3 mimetics, possibly through mPTP opening and/or MOMP. While the mechanistic and molecular links between MMP, mPTP opening, and MOMP are still not fully understood^22,27,28,79^, it has been reported that mitochondrial uncouplers promote Smac/DIABLO release from mitochondria to the cytosol, mediated by mPTP opening^80^.

Our findings have important implications for better management of the toxicity of senolytics in vivo. In combination with the uncoupler BAM15, a 0.5 mg/kg/day dose of the BH3 mimetic Navitoclax was sufficient to rescue radiation-induced premature frailty and cognitive function in mice. Typical doses of Navitoclax used in mice are in the order of 50 mg/kg/day (Suppl. Fig. S4-1), indicating that a dose reduction by two orders of magnitude can be achieved in vivo by combination with an uncoupler. In clinical trials of patients with lymphoid tumors, at least 40% of patients reported thrombocytopenia at a dose of about 3–4 mg/kg/day^5,6^ This is similar to what has been reported in rodents, and higher doses of Navitoclax have shown increased pulmonary hypertension^8^ and bone loss in aged mice^9^. Given that in rodents, human equivalent drug doses have been suggested to be around 12 times as high^81^, a corresponding dose reduction, especially if only given for a short period (in our study, 2×5 days was sufficient for an efficient and long-lasting reduction), could potentially result in significantly fewer side effects. Among mitochondrial uncouplers, BAM15 has an excellent safety profile in vivo, appears to cross the blood-brain barrier as it accumulates in the brain among other organs^53^, and has shown potential in treating metabolic disorders and protecting organs from damage^51–53,82,83^.

Our results may not only be relevant in the context of antiaging interventions but also with respect to tumor chemotherapy and the senolysis of both cancer and non-cancer cells in therapyinduced senescence. It is well established that many cancer cells show mitochondrial dysfunction with low MMPs and thus rely primarily on glycolysis, and drugs that lower MMP have been developed^84^. We show that therapy-induced senescent cancer and non-cancer cells can also be (further) sensitized by mitochondrial uncouplers.

A limitation of our study is that cell type-specific senolytic activity was not comprehensively addressed. In general, senolytics are active only in some cell types but not in others. This can be rectified to some extent by combining senolytic drugs, as in the case of Dasatinib (senolytic for preadipocytes but not HUVECs) and Quercetin (senolytic for HUVECs but not preadipocytes)^47^. We have established senolytic activity and specificity in multiple types of fibroblasts after different senescence inducers, as well as in leukemia and glioma cells in vitro. While the specificity (or not) for a wider spectrum of cell types still needs to be addressed, our in vivo results showing rescue of such complex phenotypes as frailty and short-term memory by the combination of low Navitoclax and BAM15 are encouraging. It is possible that a reduction in senescence in fibroblasts across various organs could have benefited tissue microenvironments, as fibroblasts are a predominant cell type in the stroma.

We have previously found that both D+Q and Navitoclax resulted in similar beneficial outcomes for multiple aging phenotypes in parallel experiments in vivo, despite these drugs having different cell type specificities^39^. In fact, recent results indicate that local, cell-type-specific senolysis can be less effective than systemic senolysis, even in the targeted organ^85^. The beneficial effects of senolysis on brain function are thought to be largely due to the systemic reduction of senescent cells and their SASP^86^. Accordingly, senolytic drugs which are optimized for systemic rather than cell-type-specific targeting could bring a wide range of benefits in vivo, and the BH3 mimetic and mitochondrial uncoupler combinations might be strong candidate leads.

## Supplementary Material

Supplemental information can be found online at https://doi.org/10.59368/agingbio.20240022.

Supplementary Materials

## Figures and Tables

**Table 1:** Conditions for immunohistochemistry (IHC) and immunofluorescence (IF).

Tissue	Slide Thickness (μm)	Technique	1° Antibody	Cat. #, Vendor	Dilution	2° Antibody	Cat. #, Vendor	Dilution	Detection
Brain	10	IHC	Iba1	ab178846, Abcam	1:2000	Biotinylated Goat anti-rabbit	BA-1000, Vector labs	1:200	VECTASTAIN ABC-HRP Kit, NovaRED, Vector labs
Brain	10	IF	Doublecortin	#14802, Cell signaling	1:1000	Alexa Fluor 594, Goat Anti-Rabbit	ab150080, Abcam	1:1000	n/a

## Data Availability

Datasets related to this article can be found at https://data.mendeley.com/datasets/2chc5kgx7y/1, an open-source online data repository hosted at Mendeley Data.
